# The Relationship between Tuberculosis and Influenza Death during the Influenza (H1N1) Pandemic from 1918-19

**DOI:** 10.1155/2012/124861

**Published:** 2012-07-17

**Authors:** Welling Oei, Hiroshi Nishiura

**Affiliations:** ^1^Julius Center for Health Sciences and Primary Care, University Medical Center Utrecht, 358GA Utrecht, The Netherlands; ^2^Theoretical Epidemiology, Faculty of Veterinary Medicine, University of Utrecht, 3584CL Utrecht, The Netherlands; ^3^School of Public Health, The University of Hong Kong, Level 6, Core F, Cyberport 3, Pokfulam, Hong Kong; ^4^PRESTO, Japan Science and Technology Agency, Saitama 332-0012, Japan

## Abstract

The epidemiological mechanisms behind the W-shaped age-specific influenza mortality during the Spanish influenza (H1N1) pandemic 1918-19 have yet to be fully clarified. The present study aimed to develop a formal hypothesis: tuberculosis (TB) was associated with the W-shaped influenza mortality from 1918-19. Three pieces of epidemiological information were assessed: (i) the epidemic records containing the age-specific numbers of cases and deaths of influenza from 1918-19, (ii) an outbreak record of influenza in a Swiss TB sanatorium during the pandemic, and (iii) the age-dependent TB mortality over time in the early 20th century. Analyzing the data (i), we found that the W-shaped pattern was not only seen in mortality but also in the age-specific case fatality ratio, suggesting the presence of underlying age-specific risk factor(s) of influenza death among young adults. From the data (ii), TB was shown to be associated with influenza death (*P* = 0.09), and there was no influenza death among non-TB controls. The data (iii) were analyzed by employing the age-period-cohort model, revealing harvesting effect in the period function of TB mortality shortly after the 1918-19 pandemic. These findings suggest that it is worthwhile to further explore the role of TB in characterizing the age-specific risk of influenza death.

## 1. Introduction

There have been three influenza pandemics in the 20th century, initially observed in 1918, 1957, and 1968, respectively, referred to as the Spanish (H1N1), Asian (H2N2), and Hong Kong (H3N2) influenza [1]. The most devastating pandemic known in human history is the Spanish influenza 1918-19. It has been estimated that one-third of the world population may have experienced the infection and more than 2.5% of those infected may have resulted in death [2]. Understanding the most serious pandemic and its epidemiological features is crucial for elucidating the mechanisms of severe influenza outcomes and possibly planning effective countermeasures in the future.

One of the most pressing scientific questions of the epidemiology of Spanish influenza is the atypical W-shaped curve seen in the age-specific mortality [3, 4]. Before and after the 1918-19 pandemic, the W-shape was not observed. Other pandemic and interpandemic influenza disproportionately killed infants and elderly, most commonly yielding the U-shaped (or J-shaped) age-specific mortality curve. Addressing the age-specific excess mortality estimate of the Spanish influenza pandemic using historical baseline, the deaths among the elderly tend to be diminished, but the peak among young adults still remains, suggesting an excess risk of death among those aged 25–35 years [5–7]. That is, one of the distinguishing features of the 1918-19 pandemic was the unusually high estimate of mortality among young adults.

Various explanations have been proposed to describe the observed W-shaped age-specific mortality. The existing underlying hypotheses for the W-shaped mortality distribution include the following descriptions.

- The influenza (H1N1) virus responsible for the 1918-19 pandemic was closely related to foregoing H1N1 virus(es) that might have widely circulated earlier than 1918. This could have yielded acquired immunity to the middle-aged and elderly persons [8, 9], resulting in an emphasis of mortality among naive young adults.
- Cytokine storm, that is, hyperreaction of the immune system that could potentially cause severe damages in the host, might explain the more severe outcomes observed among young adults (who have stronger immunity) and fewer deaths observed in children and elderly (who have weaker immune system) [10], although the findings have mainly stemmed from experimental studies of avian influenza in nonhuman hosts.
- From 1918-19, there was one or more underlying risk factor(s) including comorbidities that caused an elevated risk of death among young adults. Such risk factors did not significantly influence the age-specific death patterns during other pandemics and inter-pandemic influenza epidemics. As an example, it has been described that those with tuberculosis (TB) in 1918 may have been more likely to die of influenza compared with those without TB [11, 12].

In recent studies, the importance of bacterial secondary infection has been emphasized (so the hypothesis (iii) [13]), while the critical role of immunopathologic responses in determining mortality risk (hypothesis (ii)) was also highlighted [14]. The possible association between previous exposure to different influenza virus(es) and high mortality among young adults during 1918–20 pandemic was also supported by an analysis of historical data [15]. Among these, we explore (iii), with a particular focus on the relationship between TB and influenza death using a variety of epidemiological datasets. TB among young adults is specifically considered in the present study, because TB was very common and one of the leading causes of death in many countries during the early 20th century.

With respect to the role of TB in characterizing the hypothesis (iii), a study by Noymer [11] investigated the historical tuberculosis mortality in the USA, demonstrating that many people with tuberculosis were killed in 1918 leading to decreased TB mortality and transmission thereafter. His finding supported the earlier hypothesis on this subject [12, 16]. To validate the existing finding in an explicit manner, the hypothesis should be formulated by exploring different types of datasets from a variety of geographic areas and observing consistency in the findings. The present study aimed to develop a formal hypothesis that indicates TB was associated with the W-shaped influenza mortality from 1918-19. Three different types of historical data were examined, including the historical epidemiological records of influenza and TB in the USA, Japan, Switzerland, and the Netherlands. 

## 2. Materials and Methods

### 2.1. Strategy to Set Up a Hypothesis

To explore the association between TB and influenza death 1918-19, our formal hypothesis was developed through the following three major steps.

**Step 1**

First, we descriptively characterized the age-specific mortality, morbidity, and the case fatality ratio of the 1918-19 influenza pandemic. This step intended to confirm the presence of the W-shaped age-specific case fatality ratio that can indicate the presence of underlying age-specific risk factor(s) of death “given influenza” among young adults.

**Step 2**

We subsequently tested the potential univariate association between TB and influenza death using a two-by-two table based on individual datasets derived from a confined outbreak setting. We used the observational epidemiological data from a TB sanatorium.

**Step 3**

Lastly, we explicitly analyzed the epidemiological time course of TB using the age-period-cohort model. We examined how the period effect of TB mortality varied before, during, and after the Spanish influenza pandemic.

### 2.2. Epidemiological Data

**Figure 1:**
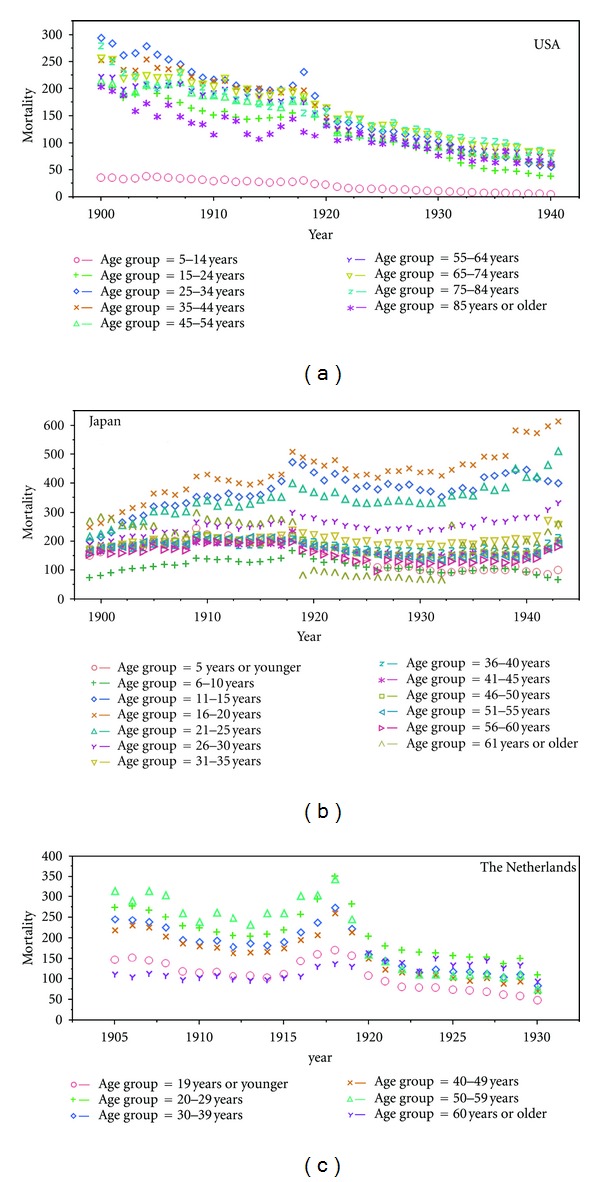
Time and age-specific tuberculosis mortality in the USA, Japan, and the Netherlands. The reported annual mortality per 100,000 persons is shown by discrete age groups.

Epidemic data of influenza cases and deaths were abstracted from published epidemic reports of the Spanish influenza pandemic in the USA and Japan [17–19]. Not only the entire USA, but also an epidemic record of Baltimore alone, was obtained [18]. For the second part of analysis, individual medical records of a Swiss TB sanatorium that experienced an influenza outbreak during the 1918-19 pandemic were extracted from the original report of the outbreak [20]. For the third analysis, TB mortality during the early 20th century was extracted from the vital statistics data in the USA, Japan, and the Netherlands [21–24] (Figure 1). Due to secondary nature of the datasets, we adhered to the original definition of cases and deaths and reporting criteria as given in the data source; for example, the case definition of influenza was not explicitly documented in the original studies [17–19] and the diagnosis was most likely based on indicator-based measure such as influenza-like illness in the present day. Influenza death was defined as a death event following influenza and pneumonia, whereas TB death was defined as the death event with any form of tuberculosis (see Section 4 for arguments on the validity). As for the TB mortality, the vital statistics data in the USA were stratified by 10-year age groups, covering the period from 1900–1940. For Japan and the Netherlands, the datasets were stratified by both gender and age, and were available from 1899–1943 for Japan and 1901–1940 for the Netherlands, respectively. Below, the mortality and morbidity data were presented per 100,000 persons following conventions.

### 2.3. Step 1: Descriptive Statistics

The mortality, the morbidity, and the case fatality ratio of the Spanish influenza were calculated in the USA and Japan. For the American datasets, Baltimore and others were separately examined due to known relatively high domination by children in non-Baltimore localities [17].

### 2.4. Step 2: Test for Univariate Association

To explore the possible influence of TB on the conditional risk of influenza death given influenza (i.e., given disease), a two-by-two table was created based on the outbreak data in the Swiss TB sanatorium. Since the comparison of mortality (i.e., deaths/population) between TB and non-TB involves a difficulty in interpreting differential risk of influenza infection and because we thus intended to examine the differential case fatality ratios between TB (cases) and non-TB individuals (controls), all subjects in the table were influenza cases. The univariate association was tested by Fisher's exact test due to small sample size. 

### 2.5. Step 3: Age, Period, and Cohort Model

To examine the period effect in TB mortality before, during, and after the Spanish influenza pandemic, we employed the age-period-cohort model to analyze the age-specific mortality data (Figure 1). Several different models were fitted to the data to explore the feasibility of decomposing the TB mortality into three effects, that is, age, period, and cohort effects. Other than the age-period-cohort altogether (APC), the alternative models included the age-only model (A), period-only model (P) and age-period model (AP). Here, we write the APC model for the log rates so that we can combine age, period, and cohort effects additively. Let *E*(*Y*_*ap*_) be the logarithms of expected mortality for age group *a* and period *p*, we described them by

(1) E(Yap)=μ+αa+βp+γc,

where *α*_*a*_, *β*_*p*_, and *γ*_*c*_ are age, period and cohort effects, and *μ* is a constant. The cohort *c* indexes diagonals of age versus period table, satisfying *c* = *p* − *a* + constant. This produces an identifiability constraint, so the model has dimension *a* + *p* + *c* − 3, one less than a full three-way factor model has. Thus, we employed one of common constraints. Suppose that *c*_1_ and *c*_2_ are two extreme cohorts in which the cohort effects are fixed; we imposed an assumption

(2) γc={δ1+β1cfor c≤c1,δ2+β2cfor c≥c2,

where *c*_1_ < *c*_2_, *δ*_1_ and *δ*_2_ are intercepts, and *β*_1_ and *β*_2_ are cohort effects in the corresponding eras, respectively [25]. Although direct interpretation of the first-order relative risk estimate is not provided, second-order changes in the slope of age, period and cohort effects can be obtained. Throughout this paper, the age, period, and cohort effects were expressed as relative risk compared with the corresponding single reference group. The age groups of 5–14 years, 0–4 years, and those younger than 20 years were selected as the reference age groups for the USA, Japan, and the Netherlands datasets, respectively. The different age grouping for the reference was due to the limited consistency of the discrete categorization between different countries. The period groups of 1900, 1899, and 1905 and the birth-year groups of 1801–1810, 1831–1835, and 1831–1840 were the reference groups for the above-mentioned three countries, respectively. Parameters were estimated by means of maximum likelihood method, assuming that each observation of TB mortality follows a Poisson distribution. We assessed the goodness of fit of the models using Akaike's Information Criterion (AIC).

## 3. Results

### 3.1. W-Shape during the Spanish Influenza Pandemic

**Figure 2:**
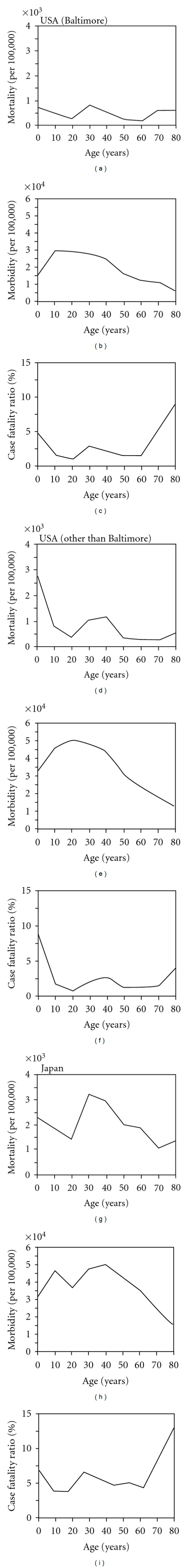
Age distributions of influenza pandemic 1918-19 in the USA and Japan. (a), (b), (c), (d), (e), (f), (g), (h), and (i) respectively, show (mortality, morbidity, and the case fatality ratio) in Baltimore, localities other than Baltimore, and Japan [17–19]. Mortality and morbidity refer to the total number of deaths per 100,000 persons and the total number of cases per 100,000 persons, respectively. The case fatality ratio is the proportion of deaths among cases.

Figure 2 illustrates how the age-specific mortality distribution was decomposed into those of the morbidity and the case fatality ratio, graphically representing the distinction between the unconditional risk of death and the conditional risk of death given influenza. Across different datasets, a consistent W-shaped distribution was observed not only in the age-specific mortality but also in the age-specific estimates of the case fatality ratio, indicating that there may have been some underlying age-specific risk factor(s) (e.g., comorbidity) that elevated the conditional risk of death among young adults.

### 3.2. Two-by-Two Table

Table 1 compares the frequency of death between TB patients (cases) and non-TB sanatorium employees (controls). All subjects in Table 1 contracted influenza during the Spanish influenza pandemic in the Swiss TB sanatorium (*n* = 88). In this sanatorium, there were 102 TB patients and 33 non-TB employees. Among 24 employee controls who contracted influenza, none died. Among 64 TB cases with influenza, 7 ended in death. The Fisher's exact test indicated that the association between TB and influenza death was marginally significant (*P* = 0.09). However, the effect size (i.e., odds ratio) was infinitely large as there was no death among influenza cases in non-TB controls.

### 3.3. Decomposition of TB Mortality

**Figure 3:**
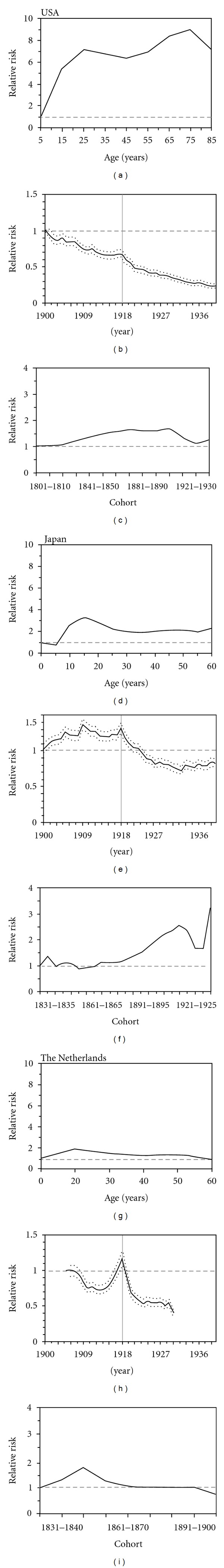
Tuberculosis mortality from 1900–1940, analyzed by age-period-cohort (APC) model in the USA, Japan, and the Netherlands. (a), (d), and (g) show the age effect, respectively, in the USA, Japan, and the Netherlands. (b), (e), and (h) show the period effect, and (c), (f), and (i) show the cohort effect for the three countries. The precisions of the age grouping were every 10 years for USA and the Netherlands, and every 5 years for Japan. The vertical solid lines and the dotted lines in (b), (e), and (h) represent the year 1918 and the 95% confidence interval (CI) of period effect derived from the profile likelihood. The dashed horizontal lines in all panels mark out the relative risk of 1.

Table 2 compares the goodness of fit of different models in describing observed TB mortality. For all the datasets, the APC model yielded the smallest AIC values for describing the TB mortality (AIC = 2735, 6081, and 1465 for the total populations of the USA, Japan, and the Netherlands, resp.).  Figure 3 shows the relative risk estimates from the APC model. The high age-effect was seen among those aged 15–25 years in all three countries, although there was also another hump among the elderly in the USA. As for the period effect, there was a consistent spike in the period effect of TB mortality for all three countries during the Spanish influenza pandemic. More importantly, the spike was followed immediately by significantly steeper decline than before the pandemic. Such tendency of period effect, which could imply that TB mortality was “washed out” by the influenza pandemic, was particularly evident in Japan and the Netherlands, but was less visible in the USA. Nevertheless, we imposed a constraint as shown in (2), and the change in the second derivative was consistently seen in three countries; so the observed patterns of period effect were consistent across countries. Cohort effect did not yield any clear consistent patterns across countries. 

## 4. Discussion

The present study examined the possible role of tuberculosis in contributing to yielding the age-specific W-shaped mortality distribution of influenza during the Spanish influenza pandemic 1918-19. The W-shaped age distribution was specific for the influenza pandemic 1918-19, and thus, we considered that the underlying reason should also be specific to 1918-19 [26, 27]. We conducted three separate analyses to implicate that TB could have been one of the plausible reasons. First, in the descriptive analysis of pandemic data, we showed that the age-specific case fatality ratio was high among young adults, indicating the possible presence of underlying risk factor(s) of death in that particular age group. Second, univariate analysis of the Swiss sanatorium data indicated that the risk of influenza death was higher among TB patients than non-TB controls. Third, the age, period, and cohort effects of TB mortality were estimated, observing possible harvesting effect not only in the mortality data but also the period function of TB mortality shortly after the influenza pandemic in Japan and the Netherlands, indicating that substantial number of TB cases died and were washed out through the pandemic. All of these findings were consistent with hypothesizing that the elevated risk of influenza death among young adults can be partially attributable to TB.

From biological and epidemiological points of view, TB can be considered to have been plausibly the underlying cause of  W-shaped risk of influenza death for several reasons. First, the age profile of the TB cases during the early 20th century (i.e., which was most commonly found among young adults) exactly overlapped the most pressing peak of the W-shaped distribution of the Spanish influenza mortality. In fact, the huge loss of life among young adults due to the Spanish influenza can be subtracted from TB mortality which was supposed to occur in the future in the absence of the Spanish influenza; that is, cohort inversion effect may have been seen [28]. In other words, an adverse event in early life (i.e., TB) may have enhanced the mortality risk from other disease (i.e., influenza). Second, tuberculosis is a chronic infectious disease that predominantly infects the same anatomical site that is affected by influenza, that is, the respiratory tract. Published studies have revealed that having influenza aggravates the pulmonary condition of TB patients, so that a closed case may become open, an arrested lesion active, or an active case progressive [29]. Although an exceptional explicit evidence stems from not the Spanish influenza pandemic but an influenza B epidemic during the mid-20th century, a historical observational study, examining the risk of worsening clinical course of TB between influenza and non-influenza patients in Denmark, clearly indicated that the clinical exacerbation of TB is induced by influenza (odds ratio = 7.05 (95% CI: 1.75, 28.39), *P* = 0.0018, Table 3) [30]. To further explore this hypothesis and offer stronger evidence than ours, not only analyzing additional epidemic data but also investigating autopsy records during the pandemic would be highly informative. Although postmortem autopsies were uncommon during the pandemic, the ascertainment of mixed infection of TB and influenza can be the direct evidence of the prevalence of mixed infection and moreover can be used for further analysis to estimate the elevated risk of influenza death among TB patients.

Of course, our historical epidemiologic study involved a number of possible biases and errors. First, the descriptive statistics of influenza cannot avoid misclassification and underascertainment, and indeed, the limited specificity of influenza-like illness is very well known [31, 32]. Second, the data from the Swiss TB sanatorium was examined only by univariate analysis, and we were not able to make any adjustments due to limited information from the individual records. The findings from such dataset are of course prone to the bias including confounding effect, especially considering that the original data stratified TB and non-TB by patients and employees (e.g., age and nutritional status can differ between cases and controls). Third, death registry was also imperfect during the early 20th century. Unlike other diseases which permit clinical confirmatory diagnosis (e.g., measles [33]), the diagnosis of TB was presumably established around the pandemic, mainly based on apparent clinical signs and symptoms of TB (e.g., hematosputum) due to very limited laboratory methods [34]. This uncertainty might have caused some of the TB patients to be misclassified as non-TB and vice versa, which could have led to biased estimate of TB mortality in the population.

It should be noted that our hypothesis does not refute any other hypotheses that have been put forward to explain the W-shaped age-specific mortality pattern. Both the pre-existing immunity and cytokine theory remain to be Plausible and in fact are consistent with a part of our results including descriptive statistics in Figure 2. Nevertheless, the preexisting immunity involves a well-known paradox that has yet to be answered: an obscure precursor virus that left no detectable trace today would have had to have appeared and disappeared before 1889 and then reappeared more than three decades later [2]. As for the cytokine theory, the precise reason for selectively observing cytokine storm in the lung among young adults has yet to be fully clarified, but a recent study identified selective pathogenic responses among young adults [14]. Moreover, rather than attributing our results to TB, there can be another hypothesis formed from our results: the atypical W-shaped feature in the case fatality ratio could have been influenced by the World War I from 1914–18. That is, poor nutrition and low socioeconomic status due to the war could have influenced both TB and the risk of influenza death among young adults. This point may also hamper the strength of our finding on the period effect of TB mortality due to a mixed effect of the war and the pandemic. Nevertheless, we partly addressed the issue of the World War I by examining a historically “neutral” country, that is, the Netherlands, which was not technically involved in the World War I (though one should note that the period effect in the Netherlands shows a start of surge prior to the 1918 pandemic, which is likely to have been caused by war).

Despite the presence of various potential explanations for the W-shaped age-specific influenza mortality 1918-19, our study has firmly and consistently presented multiple findings in a systematic fashion, implicating that TB was associated with observing the W-shaped mortality distribution of influenza. What are the practical implications in the present day from answering this question? Should a highly fatal influenza pandemic occur in the future [35], testing the role of TB in characterizing the risk of death would be extremely useful in minimizing the disaster, because TB is still prevalent in many developing countries and the transmission dynamics are known to be highly heterogeneous and very slow [36]. If TB cases appear to be at a particularly high risk of influenza death, one could consider targeted prevention (e.g., prioritized vaccination) and close monitoring of cases as well as early diagnosis and treatment to improve the clinical outcome. Further investigations of the association between TB and influenza death for both pandemic and interpandemic influenza could shed light on identifying this important risk group of death in the population.

## Figures and Tables

**Table 1:** Cross tabulation of tuberculosis and conditional risk of death among influenza cases in a Swiss tuberculosis sanatorium, 1919 (*n* = 88).

	Dead	Survived
Tuberculosis patients	7	57
Employee (non-tuberculosis)	0	24

Fisher's exact test: *P* = 0.09.

**Table 2:** Different model fit for describing tuberculosis mortality in the USA, Japan, and the Netherlands.

Country	Gender	Model	Number of parameters	d.f.	AIC
USA [21]	Total	A	9	360	9,681
		P	41	328	9,823
		AP	49	320	2,884
		APC	63	306	2,735

Japan [22, 23]	Female	A	13	572	8,907
		P	45	540	37,098
		AP	57	528	7,270
		APC	80	505	5,758
	Male	A	13	572	16,358
		P	45	540	33,924
		AP	57	528	14,471
		APC	80	505	9,845
	Total	A	13	572	9,429
		P	45	540	28,800
		AP	57	528	8,207
		APC	80	505	6,081

The Netherlands [24]	Female	A	6	150	3,345
		P	26	130	2,984
		AP	31	125	1,748
		APC	40	116	1,467
	Male	A	6	150	4,095
		P	26	130	3,860
		AP	31	125	1,860
		APC	40	116	1,500
	Total	A	6	150	3,685
		P	26	130	3,292
		AP	31	125	1,763
		APC	40	116	1,465

Note: Each row shows the results from a single model of tuberculosis mortality for the period from 1900–1940, with the corresponding number of parameters and the degree of freedom (d.f). A: age model; P: period model; AP: age-period model; APC: age-period-cohort model. The goodness-of-fit test is assessed using AIC (Akaike's Information Criterion) calculated from each model.

**Table 3:** Cross tabulation of clinical course of tuberculosis and influenza B infection in a Danish tuberculosis sanatorium, 1953 (*n* = 195).

	Clinical course	
	Worsened	Unchanged
Influenza	7	46
No influenza	3	139

Chi-square test: *P* = 0.0018.
